# Impact of Bariatric and Metabolic Surgery on Sarcopenia-Related Parameters According to the EWGSOP2 Consensus Criteria in Persons Living with Obesity

**DOI:** 10.1007/s11695-025-07816-6

**Published:** 2025-03-31

**Authors:** Paulo Cardoso, Tânia V. Santos, Marta Ramon-Krauel, Sandra Pais, Ana Luísa De Sousa-Coelho

**Affiliations:** 1 Unidade Local de Saúde do Algarve (ULSALG), Unidade de Faro, Rua Leão Penedo, Serviço de Cirurgia 1, 8000-286 Faro, Portugal; 2 Faculdade de Medicina e Ciências Biomédicas (FMCB), Universidade do Algarve (UAlg), Campus de Gambelas, 8005-139 Faro, Portugal; 3 Faculdade de Ciências e Tecnologia (FCT), Universidade do Algarve (UAlg), Campus de Gambelas, 8005-139 Faro, Portugal; 4 Department of Endocrinology, Institut de Recerca Sant Joan de Déu, Hospital Sant Joan de Déu, Barcelona, Spain; 5 Universidade de Évora (UE), Comprehensive Health Research Centre (CHRC), Rua Romão Ramalho 59, 7002-554 Évora, Portugal; 6 Escola Superior de Saúde (ESS), Universidade do Algarve (UAlg), Campus de Gambelas, 8005-139 Faro, Portugal; 7 Algarve Biomedical Center Research Institute (ABC-Ri), Universidade do Algarve (UAlg), Campus de Gambelas, 8005-139 Faro, Portugal

## Abstract

Although bariatric and metabolic surgery (BS) has proved effective in the treatment of obesity based on the reduction in fat mass and the remission of comorbidities, there is also loss of lean mass after BS which could compromise muscle functionality. According to the European Working Group on Sarcopenia in Older People (EWGSOP), sarcopenia is a disease associated with loss of muscle mass, strength, and function. Through a comprehensive review of the literature, we identified a range of studies focusing on evaluating sarcopenia-related parameters according to the EWGSOP2 consensus criteria, before and after BS. Although most studies reported reductions in skeletal muscle mass and absolute muscle strength after surgery, improvements in muscle functionality were generally achieved, independent of the type of BS.

## Introduction

Obesity is a complex and multifactorial condition and is considered a major global public health problem, as it is a risk factor for the development of chronic non-communicable diseases, that in the long term reduce both quality of life and longevity [1, 2]. Although several treatment options are available, the literature shows that bariatric and metabolic surgery (BS) is currently the most effective treatment option to reach the goal of sustainable weight loss and the resolution of associated chronic pathologies [3, 4]. Bariatric surgery provides substantial weight loss, causing both the loss of fat mass and muscle mass [5–7].

After BS, patients may be more susceptible to deficiencies in macronutrients (e.g., proteins) and micronutrients (e.g., vitamin D, magnesium), which can trigger or aggravate already existing pathologies, such as osteoporosis, anemia or excessive muscle mass loss [8–10]. Consequently, there is a potential risk of worsening pre-existing sarcopenic obesity or inducing the onset of sarcopenia. This risk might be greater in the first months after the surgery when there might occur greater loss of muscle mass and function [6, 11]. However, the metabolic effects of these changes are not well documented.

Sarcopenia has been challenging to define over the years [12, 13]. In 2016, sarcopenia was awarded an International Classification of Diseases-10th version (ICD-10) diagnosis code [M62.84], to allow for better case identification, screening, and diagnosis of sarcopenia [14, 15]. The European Working Group on Sarcopenia in Older People (EWGSOP) described sarcopenia as a “progressive and generalized skeletal muscle disorder associated with increased likelihood of hostile outcomes such as falls, fractures, physical disability, and mortality” [16]. Initially, sarcopenia was solely described in the elderly population as the age-associated loss of muscle mass and strength [17]. However, it is currently accepted that there are several causes that give rise to this disease and that it can occur earlier in adulthood [18]. Additionally, sarcopenia is associated with other diseases such as metabolic syndrome, liver failure, cardiovascular diseases (CVD), and osteoporosis [19–24].

Sarcopenic obesity (SO) is defined as a clinical condition in which an individual, characterized by having excess adiposity, has reduced lean body mass [25–27]. Similar to sarcopenia, there is no universal consensus for the definition, diagnostic criteria, or the threshold values for the diagnosis of SO [26, 27]. For that reason, the prevalence of SO may vary depending on the definitions applied [28]. For the identification of SO, the European Association for the Study of Obesity (EASO) and the European Society for Clinical Nutrition and Metabolism (ESPEN) recommends considering the presence of three components: elevated body mass index (BMI) or waist circumference, low muscle mass, and low skeletal muscle function (for instance, muscle strength) [27], acknowledging that future research is still necessary to determine the optimal cut-off points for SO (Table 1).

**Table 1:** Sarcopenia diagnostic criteria and cut-off points (when available), according to different working groups

	***Low muscle strength***	***Low muscle mass***	***Low physical performance***
**EASO/ESPEN** [27]	**Handgrip strength (HGS)** **Knee extension strength** **5-times chair stand time (5 × SST)** **30 s chair stand test (30 s SST)**	**Body composition: DXA, BIA, CT**	Not considered
**EWGSOP2** [16]	**Handgrip strength** M: < 27 kg F: < 16 kg **5-times chair stand time** M/F: ≥ 15 s	**BIA and DXA:** **ASM** M: < 20 kg F: < 15 kg **ASM/height**^**2**^ M: < 7.0 kg/m^2^ F: < 5.5 kg/m^2^	**Gait speed (GS) test:** M/F: < 0.8 m/s **SPPB:** M/F: score ≤ 8 **Timed-Up and Go test (TUG):** M/F: ≥ 20 s **400-m walk test:** Non-completion or ≥ 6 min for completion
**AWGS** [29]	**Handgrip strength:** M: < 28 kg F: < 18 kg	**BIA (ASM/height**^**2**^**):** M: < 7.0 kg/m^2^ F: < 5.4 kg/m^2^ **DXA scan (ASM/BMI)** M: < 0.789 kg/BMI F: < 0.512 kg/BMI	**6MWT:** M/F: < 1.0 m/s **SPPB:** M/F: score ≤ 9 **5-times chair stand time (5 × SST):** M/F: ≥ 12 s
**FNIH** [30]	**Handgrip strength:** M: < 26 kg F: < 16 kg	**DXA scan (ASM/BMI)** M: < 0.789 kg/BMI F: < 0.512 kg/BMI	**Gait speed (GS) test:** M/F: < 0.8 m/s
**IWGS** [31]	Not considered	**DXA scan (ALM/ height**^**2**^**)** M: ≤ 7.23 kg/m^2^ F: ≤ 5.67 kg/m^2^	**Gait speed (GS) test:** M/F: < 1.0 m/s

*EASO*, European Association for the Study of Obesity; *ESPEN*, European Society for Clinical Nutrition and Metabolism; *EWGSOP2*, European Working Group on Sarcopenia in Older People; *AWGS*, Asian Working Group for Sarcopenia; *FNIH*, Foundation for the National Institutes of Health; *IWGS*, International Working Group on Sarcopenia; *BIA*, Bioelectrical Impedance Analysis; *DXA*, dual-energy X-ray absorptiometry; *CT*, computerized tomography; *BMI*, body mass index; *ASM*, appendicular skeletal mass; *ALM*, appendicular fat lean mass; *SPPB*, Short Physical Performance Battery; *6MWT*, six-minute walk test

The revised EWGSOP consensus (EWGSOP2) considers *muscle strength* to be the main parameter for identifying sarcopenia, considering it as the most reliable measure of skeletal muscle function, rather than muscle mass [16]. The second criterion is then the *quality or quantity of muscle*, and the third criterion is *physical performance* [16] (Table 1). In addition to EWGSOP, EASO, and ESPEN, other associations or working groups such as the Asian Working Group for Sarcopenia (AWGS) [29], the International Working Group on Sarcopenia (IWGS) [31], or the Foundation for the National Institutes of Health Biomarkers Consortium Sarcopenia Project (FNIH) [30], have their own definitions of sarcopenia, meaning that there is still no universal consensus on the tests performed or the diagnostic threshold values (Table 1). Indeed, only by combining the many different parameter cutoffs proposed in the EASO/ESPEN definitions [32], several definitions of sarcopenic obesity could be possible [33]. Given these definitions, we considered the effect on all sarcopenia-related parameters as defined by the EWGSOP2 consensus criteria, including the evaluation of physical performance, muscle mass, and strength.

There are significant challenges in the diagnosis of sarcopenia, particularly with regard to establishing diagnostic tools and consistent criteria [34]. With this review, we seek to elucidate the potential impact of BS for the treatment of obesity, on sarcopenia, as defined as an accelerated loss of skeletal muscle mass associated with decreased functional capacity. Because focusing solely on either muscle mass or muscle strength can be misleading, we considered the effect on the sarcopenia-related parameters as defined by the EWGSOP2 consensus criteria: physical performance, muscle mass, and strength.

## Methods

This is a non-systematic review, based on a literature search of the US National Library of Medicine catalog (PubMed), using selected search terms (Table 2). A first search was performed from February 4, 2022, to July 31, 2022, and then from February 6, 2023, to March 21, 2023. A final confirmatory search was performed on June 12, 2024.

**Table 2:** Search strategy with the defined keywords in combination

General keywords		Specific keywords
(Bariatric Surgery[Title/Abstract]) OR (Metabolic Surgery[Title/Abstract]) OR (Gastric Bypass[Title/Abstract]) OR (Roux-en-Y[Title/Abstract]) OR (RYGB[Title/Abstract]) OR (Sleeve Gastrectomy[Title/Abstract])	AND	(sarcopenia) OR (sarcopenic) OR (EWGSOP)
		(muscle strength) AND (gait speed)
		(muscle strength) AND (physical performance) AND ((lean mass) OR (muscle mass))

The inclusion criteria for the first selection were longitudinal studies with at least one evaluation of either body composition, muscle strength or physical performance, both before and at least at once, at least 1 month, after BS. The second selection included only those studies with all three types of evaluation (physical performance, muscle mass, and strength) for the diagnosis of sarcopenia according to the EWGSOP2. Cross-sectional studies were considered only if all three types of evaluations were available and if a non-operated control group (i.e., patients with obesity) existed. Studies that evaluated exercise or supplementation protocols and did not have a non-exercised/supplemented group of patients (i.e., a true control/placebo group, without intervention) were excluded. Case reports were not considered.

## Results

### Characteristics of the Studies

From the literature search of PubMed, using several combinations of keywords related to sarcopenia (Table 2), a total of 159 unique references were obtained. A total of 44 original articles were preliminarily selected. From these, 17 studies only evaluated either the body composition or muscle strength, while 1 only evaluated physical performance, before and 2 months after BS [35]. Many others also failed to include all three parameters—muscle mass, muscle strength, and physical performance—as required by the EWGSOP2 (Table 3, not highlighted).

From this first screening, a total of 54 Reviews/Editorials/Letters/Consensus/Guidelines were identified (not shown). From these, 3 were recent systematic reviews with meta-analysis, where the authors considered the variables *muscle mass* and *muscle strength*, but not changes in *physical performance* after bariatric and metabolic surgery [36–38]. In addition, a total of 6 published Protocols were identified. These included evaluating the effect of supervised exercise training, or a specific supplement administration, to decrease the risk of sarcopenia after BS, and proposed assessing the levels of myokines, insulin resistance and patients’ quality of life, among other measurements [39–44]. From the studies included in Table 3, only 9 provided a definition for sarcopenia or the thresholds for the diagnostic tests used.

For the 9 studies selected (Table 3, highlighted in bold) [45–53], there were a variety of tests performed for each item (Table 1), and many different methodologies were used to identify changes in muscle mass, muscle strength and physical performance. Six were longitudinal studies (Table 4), and the paired evaluations were performed at varied timepoints related to the surgery (Table 3).

**Table 3:** Identification of studies that either defined sarcopenia or evaluated at least two sarcopenia-related parameters

Author, year	Definition of sarcopenia or sarcopenic-obesity parameters	Muscle strength	Body composition	Physical performance	Number of patients (type of BS)	Follow-up time after BS	Ref
Orioli, 2024	BMI ≥ 30 kg/m^2^ and/or FM ≥ 25% (male), ≥ 35% (female) and SMM/BW < 37.7% (male), < 26.7% (female)	HGS	BIA	*nd*	Total = 62 (SG = 39; RYGB = 23)	m0, m3	[54]
Schiavo, 2024	Not provided	HGS	BIA	*nd*	Total = 57 (SG)	m0, m1	[55]
Boppre, 2024	Not provided	Knee/trunk extension/flexion	DXA	*nd*	Total = 61 (SG and RYGB)	m0, m1, m6, m12	[56]
Gadducci, 2024	Not provided	Knee extension/flexion	BIA	*nd*	Total = 123 (RYGB)	m0, m6, m36	[57]
**Crispim Carvalho, 2023**	ASM/wt and/or HGS in the lowest quartile of the sample	HGS	BIA, DXA	6MWT	Total = 36 (SG = 9; RYGB = 27)	m0, m3, m6, m12	[45]
**Florêncio, 2023**	EWGSOP criteria [58]	HGS	BIA, DXA	GS6m	Total = 69 (RYGB = 30)	cross-sectional	[46]
Crispim Carvalho, 2023	ASM/wt and/or HGS in the lowest quartile of the sample	HGS	BIA	*nd*	Total = 34 (SG and RYGB)	m0, m3, m6, m12	[59]
Ruthes, 2022	FNIH classification criteria and EWGSOP2 consensus	HGS	DXA	*nd*	Total = 28 (RYGB)	m0, m6, m12	[60]
**Buzza, 2022**	EWGSOP2 (LLM with low strength)	HGS; SST	DXA	GS4m; SPPB	Total = 120 (RYGB = 60)	cross-sectional	[47]
Zhou, 2022	Not provided	HGS; knee extension	DXA	Aerobic capacity	Total = 13 (SG = 12; RYGB = 1)	m0, m6	[61]
**Gil, 2021**	Not provided	leg- & bench-press; SST	DXA	TUG	Total = 55 (RYGB)	m0, m3, m9	[48]
Diniz-Sousa, 2021	Not provided	knee extension	DXA	*nd*	Total = 61 (SG and RYGB)	m0, m1, m6, m12	[62]
Gerken, 2021	Not provided	HGS	BIA	*nd*	Total = 198 (SG = 68; RYGB = 130)	m0, w6, m3, m6, m12, m24	[63]
**Coral, 2021**	EWGSOP2 (low HGS, GS, TUG)	HGS	BIA	TUG, GS4m	Total = 62 (SG = 41; RYGB = 21)	m0, m6	[49]
Reinmann, 2021	Not provided	5xSST, quadriceps strength	*nd*	GS, 6MWT	Total = 33 (RYGB)	m0, m3	[64]
**de Oliveira, 2020**	Not provided	HGS	BIA; DXA	GS4m	Total = 73 (SG = 20; RYGB = 16)	cross-sectional	[50]
**Alba, 2019**	Not provided	HGS; 5xSST	DXA	GS4m, 400mWT	Total = 47 (RYGB)	m0, m6, m12	[51]
Oppert, 2018	Not provided	HGS; 1RM	DXA	*nd*	Total = 76 (RYGB)	m0, m6	[65]
Voican, 2018	SMI < 52.4 cm^2^/m^2^ (male) and < 38.5 cm^2^/m^2^ (female)	*nd*	CT	*nd*	Total = 184 (SG)	m0, m12–18	[66]
Hassannejad, 2017	Not provided	60 s-SST; 1RM	BIA	12MWRT	Total = 60 (SG = 33; RYGB = 27)	m0, m3	[67]
Campanha-Versiani, 2017	Not provided	10RM	DXA	*nd*	Total = 37 (RYGB)	m0, m9, m12	[68]
Cole, 2017	Not provided	HGS	DXA	*nd*	Total = 5 (RYGB)	m0, m1.5, m6, m12, y9	[69]
Schollenberger, 2016	Criteria described by Baumgartner [13]	HGS	BIA	*nd*	Total = 20 (SG = 15; RYGB = 5)	m0, m1, m3, m6	[70]
Otto, 2014	Not provided	HGS	BIA	*nd*	Total = 25 (SG and RYGB)	m0, w6, w12, m4	[71]
**Stegen, 2011**	Not provided	HGS; 1RM; 30 s-SST	BIA	6MWT	Total = 15 (RYGB)	m0, m4	[52]
**Miller, 2009**	Not provided	5xSST; knee extension	BIA	SPPB; GS4m;	Total = 28 (RYGB)	m0, w3, m3, m6, m12	[53]

*BS*, bariatric and metabolic surgery; *SG*, sleeve gastrectomy; *RYGB*, Roux-en-Y gastric bypass; *SO*, sarcopenic obesity; *HGS*, handgrip strength; *BIA*, bioelectrical impedance analysis; *CT*, computed tomography; *DXA*, dual-energy X-ray absorptiometry; *SMM*, Skeletal Muscle Mass; *BW*, body weight; *LLM*, low lean mass; *ASM/wt*, appendicular skeletal mass adjusted for weight; *SMI*, skeletal muscle index; *GS*, gait speed; *6MWT*, six-minute walk test; *2MWT*, two-minute walk test; *12MWRT*, twelve-minute walk-run test; *SST*, sit-to-stand test; *TUG*, timed-up and go test; *1RM*, one repetition maximum test; *10RM*, ten repetition maximum test; *SPPB*, short physical performance battery; *m*, month; *w*, week; *y*, year; *nd*, not determined

Three of the articles included described cross-sectional studies [46, 47, 50] (Table 5). Although ranging from 2009 to 2023, most studies were published in the last decade, indicating a relatively recent interest in this subject. The authors focused on different specific main goals, such as evaluating the prevalence of sarcopenia after the surgery [47], correlating pre-existing sarcopenia with metabolic outcomes post-surgery [45, 49], evaluating the physical function improvements [53], or the effects of exercise on muscle remodeling and physical fitness, following BS [48, 52]. Six studies were performed in Brazil [45–50], 1 in Belgium [52], and 2 in the United States of America (USA) [51, 53] (Table 4, Table 5).

**Table 4:** Baseline characterization of the population of the longitudinal studies that evaluated muscle strength, body composition and functional capacity in patients before and after bariatric and metabolic surgery

Author, year	N. individuals	Age (years)	Sex (% female)	BMI (Kg/m^2^)	Country	Ref
Crispim Carvalho, 2023	*n* = 36	~ 39.8*	100	~ 42.5*	Brazil	[45]
Gil, 2021	*n* = 40 ^#1^	42 ± 8	100	47.4 ± 7.6	Brazil	[48]
Coral, 2021	*n* = 62	38.4 ± 10.8	83.9	42.2 ± 5.4	Brazil	[49]
Alba, 2019	*n* = 47	45 ± 12	79	44 ± 7	USA	[51]
Stegen, 2011	*n* = 7	43.1 ± 5.6	75	40.4 ± 8.1	Belgium	[52]
Miller, 2009	*n* = 28 ^#2^	43.5 (27.1–59.1)	92.9	53.0 ± 1.6	USA	[53]

*BS*, bariatric and metabolic surgery; *BMI*, body mass index; *USA*, United States of America; *Calculated value based on weighted average. ^#1^ Only 27 from the 40 patients considered at baseline were submitted to BS. ^#2^ Only 19 from the 27 patients that were actually submitted to BS were included in the subsequent analysis

**Table 5:** Characterization of the populations of the cross-sectional studies that evaluated muscle strength, body composition, and functional capacity in a group of patients after bariatric and metabolic surgery (BS) and in a control group (Ctl)

Author, year	Group	N. individuals	Age (years)	Sex (% female)	BMI (kg/m^2^)	Country	Ref
Florêncio, 2023	BS	*n* = 30	39.1 ± 7.19	66.7	27.1 ± 3.51	Brazil	[46]
	Ctl	*n* = 39	38.1 ± 8.81		44.2 ± 5.47		
Buzza, 2022	BS	*n* = 60	50.3 ± 9.7	100	30.2 ± 4.8	Brazil	[47]
	Ctl	*n* = 60	50.2 ± 9.7		35.5 ± 5.6		
de Oliveira, 2020	BS	*n* = 36	41.4 ± 12	94.4	34.8 ± 6.9	Brazil	[50]
	Ctl	*n* = 37		*ns*	42.9 ± 5.7		

*BS*, bariatric and metabolic surgery group; *Ctl*, control group; *BMI*, body mass index; *ns*, not shown

### Assessment of Sarcopenia According to the Ewgsop2 Pre- and Postoperatively

#### Longitudinal Studies

**Fig. 1:**
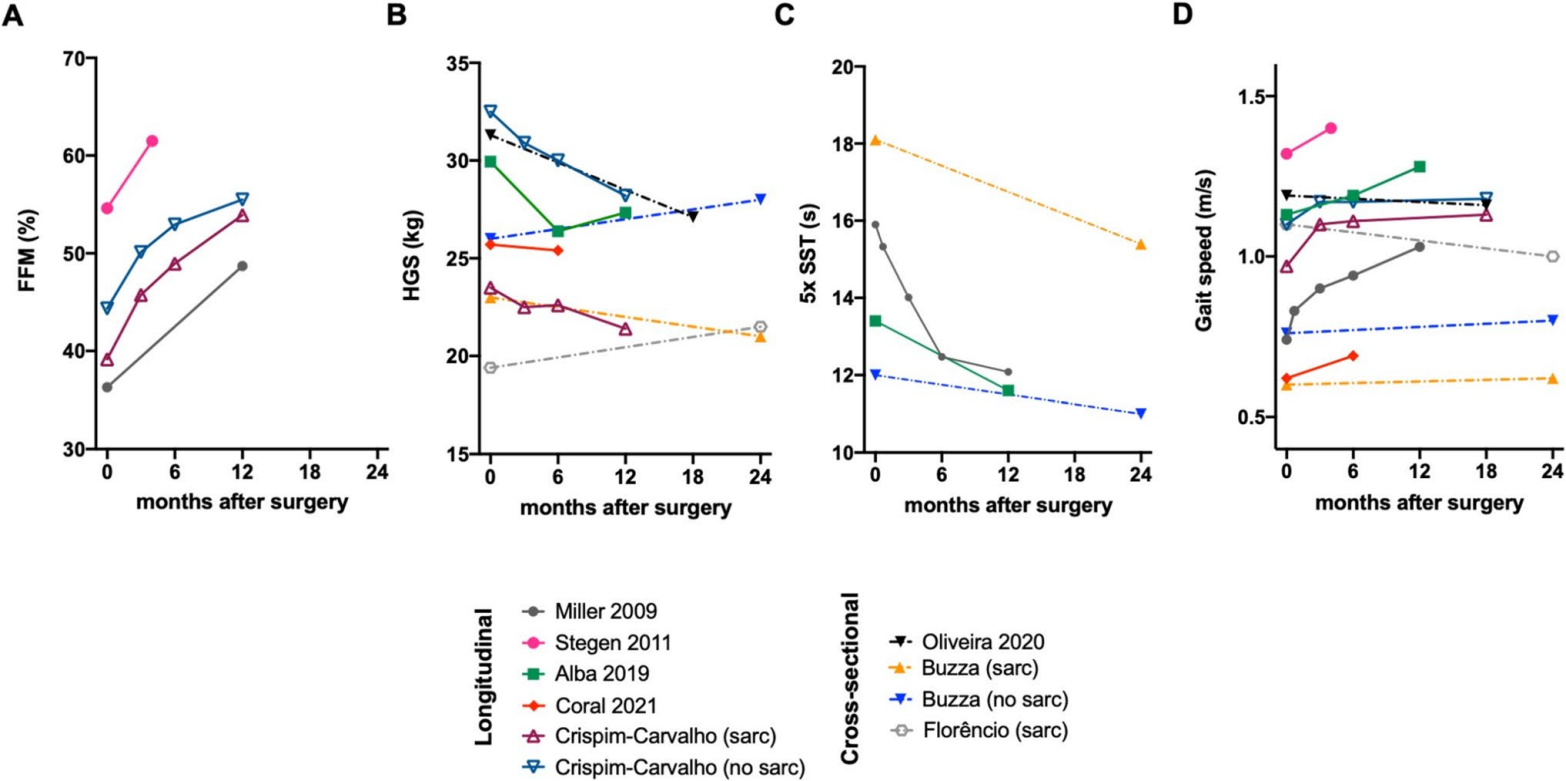
Baseline and postoperative follow-up data of the different diagnostic criteria to define sarcopenia according to the EWGSOP2 recommendations at month 0 and different months after surgery. **A** Fat-free mass (FFM) represented as % to body weight (BW); **B** hand grip strength (HGS) (kg); **C** five times sit-to-stand test (5x-SST) (s); **D** usual gait speed (GS) test (m/s). Data were extracted from articles by Miller et al. [53], Stegen et al. [52], Alba et al. [51], Coral et al. [49], Crispim-Carvalho et al. [45], Oliveira et al. [50], Buzza et al. [47], and Florêncio et al. [46]. When comparing a control group (represented at m0) with surgical patients 18 or 24 months after surgery, dashed lines were used. When patients were classified with or without sarcopenia, the terms “sarc” and “no sarc” were used, respectively

Out of the studies forementioned (Table 3), only 6 assessed the 3 sarcopenia-related parameters (muscle mass, muscle strength, and physical performance) defined by the EWGSOP2, longitudinally (Table 6) [45, 48, 49, 51–53]. To the best of our knowledge, the study by Miller and colleagues [53] was the first to evaluate the impact of the weight loss induced by surgical treatment of obesity in the physical function of the patients, including the assessment of body composition, muscle strength, and functionality. The authors showed a decrease in the absolute fat-free mass (FFM) of the patients 1 year after BS, although the relative FFM (normalized to BW) increased [53]. This was a common feature observed in the included studies (Fig. 1A), highlighting that although lean mass is lost, the greater proportion of body mass loss is due to the loss of fat mass. These results were independent of the body composition being evaluated by BIA or DEXA (Table 6).

**Table 6:** Sarcopenia-related parameters from patients at different follow-up visits after bariatric surgery (longitudinal studies)

Author, year	Muscle strength (absolute)			Body composition (absolute FFM, lean mass, SMM)		Physical performance		Ref
	**HGS**	**Other**	**SST**	**BIA**	**DXA**	**GS**	**TUG**	
Crispim Carvalho, 2023	Decreased m12	-	-	Decreased	*ns*	Improved	-	[45]
Gil, 2021	-	Decreased m3 ^(1)^	Improved m9	-	Decreased m3-m9	-	Improved m9	[48]
Coral, 2021	Unchanged m6	-	-	Decreased m6	-	Improved m6	Improved m6	[49]
Alba, 2019	Decreased	-	Improved m12	-	Decreased m12	Improved m12	-	[51]
Stegen, 2011	Decreased m4	Decreased ^(2)^	Unchanged m4	Decreased m4	-	Unchanged	-	[52]
Miller, 2009	-	Decreased m6-m12 ^(3)^	Improved w3-m12	Decreased m12	-	Improved m3-m12	-	[53]

^(1)^ lower- and upper-limb strength (1-RM leg- and bench-press tests); ^(2)^ dynamic strength of the quadriceps, hamstrings, biceps and triceps (1RM); ^(3)^ maximal torque in the isometric knee extension at 90°. *FFM*, fat-free mass; *SSM*, skeletal muscle mass; *HGS*, handgrip strength; *SST*, sit-to-stand test; *BIA*, bioelectrical impedance analysis; *DXA*, dual-energy X-ray absorptiometry; *GS*, gait speed; *TUG*, timed-up and go test; *w*, week; *m*, month; *ns*, not shown

Most studies evaluate muscle strength by using a dynamometer that measures hand grip strength (HGS), although some also assessed lower limb strength. In the study of Miller et al., the quadriceps strength was decreased when expressed on an absolute basis (Table 6). However, when expressed on a relative scale (normalized by BW), improvements were found 6 months after BS and maintained over 1 year [53]. The absolute muscle strength remained unchanged in only one of the studies, from baseline to 6 months after BS [49], while in all other longitudinal studies it significantly decreased (Fig. 1B; Table 6). Due to the greater proportional reduction in body weight compared to the decrease in strength, relative muscle strength increased. Regarding specific functional tests, which also reflect lower limb muscle strength, patients improved their results in the timed sit to stand (SST) in the early postoperative period (3 weeks after BS) [53] and continued improving (i.e., decreased time in performing the test) until the 1-year follow-up visit (Fig. 1C), except in the Stegen et al. study [52], evaluated 4 months after BS (not shown). In these individuals, the gait speed (GS) was only marginally improved (Fig. 1D), not reaching statistical significance [52]. This was in contrast with the results of all other studies where GS showed statistically significant improvements, at the 3-week visit after BS [53], and at 6, 12, or even 18 months postoperatively (Fig. 1D). Patients also showed improvements in additional tests, namely Timed Up and Go (TUG) and 400-m walking test (Table 6). When exercise training programs were included [48, 52], only the data from the control arm of the study (“the untrained patients”) were considered.

Only one longitudinal study established 2 groups based on sarcopenia-related parameters in patients with obesity at baseline, where the group with sarcopenic obesity was defined as low Appendicular Skeletal Mass (ASM)/wt and/or HGS in the lowest quartile of their sample [45]. Independently of the group that patients were assigned preoperatively, all showed increased relative fat-free mass (FFM) (Fig. 1A), decreased absolute HGS (Fig. 1B) (although increased when normalized to BMI), and increased GS at the 3-, 6-, and 12-month follow-up visits after BS (Fig. 1D) (Table 6).

#### Cross-sectional Studies

All cross-sectional studies included matched the intervention group (postoperative) with a group of unoperated individuals with obesity (preoperative/controls) [46, 47, 50]. De Oliveira et al*.* compared patients after BS with a control group of patients with obesity that had surgical indications for BS as per guidelines [50]. The control group (with obesity) had higher absolute HGS (Fig. 1D), but similar GS (Fig. 1B), when compared with the intervention group (evaluated at a median of 18 months after BS). Buzza et al. studied a female population status post laparoscopic RYGB surgery [47]. Through DXA scans, muscle strength, and physical performance tests, the authors identified that, at a minimum of 2-year follow-up, a sub-group of the patients had sarcopenia [47]. These represented 28% of their sample (*n* = 17), which was higher than those identified with altered sarcopenia-related parameters in the control group (*n* = 10, 17%). When compared with the *sarcopenia control group*, the *sarcopenia after surgery group* showed better results at the short physical performance battery (SPPB) score and SST, with similar results for the HGS and GS (Fig. 1B, C, D). Finally, in another recent cross-sectional study [46], the authors identified a similar proportion of patients with HGS below the ESGWOP threshold in the postoperative group vs*.* a control (pre-op) group (i.e., a total of 6 individuals in each group). However, only 1 of the females (with low HGS) had a GS test result below 0.8 m/s, who was in the preoperative group (the control group of patients with obesity). When comparing the sarcopenia-related parameters for these 6 patients with sarcopenia in each group (post-op *vs.* pre-op), statistically significant differences were exclusively found in Appendicular Lean Mass (ALM) (kg/h^2^), but not in HGS or GS [46].

## Discussion

Muscle mass is fundamental to a healthy metabolism, as it has important functions such as thermoregulation, accumulation of fat, glycogen and proteins, bone remodeling, and preservation of the muscle function [72]. A substantial loss of muscle mass can result in functional impairment, decreased basal metabolism, and a lower quality of life for the individual [19]. However, muscle mass alone may not always represent muscle strength and functionality. For this review, we chose to evaluate studies based on the EWGSOP perspective (sarcopenia in the general population) rather than based on the ESPEN perspective (sarcopenia in individuals with obesity) because after an optimal clinical response to bariatric and metabolic surgery, most patients will not be in the obesity range. Including the evaluation of the physical performance or functional capacity with additional tests allows a better characterization of the patients both preoperatively and at early timepoints where specific nutritional and/or physical interventions can still be proposed in the context of personalized medicine. According to the EWGSOP, physical performance can be assessed, for instance by GS or TUG [16]. Nevertheless, GS can be assessed by different methods, and not only by the GS4m (time spent walking 4 m), but also with the 4- or 6-MWDT (distance walked during 4 or 6 min, respectively [73]). Importantly, TUG may be considered either a measure of functional capacity, balance, or overall mobility [74].

Although all the aforementioned studies included the evaluation of the 3 sarcopenia-related parameters, most studies did not aim to diagnose sarcopenia. This may be due to the lack of an accepted universal definition for the diagnosis of sarcopenia [75]. Indeed, although not mentioned by the authors in Alba et al., from the data reported in its figures, it is possible to envisage around 17% of individuals with a GS below 0.8 m/s [51], suggesting patients with sarcopenia could be present in their sample. From the study of Miller et al., GS at baseline was on average below the EWGSOP2 criteria, but early at 3 weeks after BS it was already slightly above [53]. In all longitudinal studies described, GS improved after BS, and only in the study by Coral et al., patients showed values below 0.8 m/s after BS [49]. This is not due to differences in the age of the patients, as this study included the youngest group from all studies. This may be explained by the type of test given (4-MWDT), or perhaps other unknown intrinsic differences from the sample of study. Regarding the improvements observed, changes in the patients’ body biomechanics may be consequentially contributing to better overall muscle function, but also the decrease in the body volume previously occupied by both muscle and fat in the patients’ legs, may allow a more comfortable walking and thus a better performance in the physical tests.

From the analysis of the data reported in the Alba et al. figures [51], we noted that over 50% of patients had a result of the timed sit-to-stand test (SST) over the proposed threshold of 15 s [16]. This is because the authors granularly plotted the individual values for each patient, while in other studies only the mean and standard deviations are available. The results from the SST showed improvements in most cases, including the cross-sectional studies and/or those that analyzed in sub-groups based on specific sarcopenia-related parameters. This means that despite the decreases in body weight and FFM observed after BS, patients improve their functional capacity and lower limb strength.

Another common measure of muscle strength is the HGS. In all the longitudinal studies included, absolute HGS decreased, but not to below the threshold proposed for females (16 kg) [16]. However, when normalized to either BW or BMI, relative HGS increased in all cases after BS, similar to what was recently reviewed [36]. Indeed, only in certain sub-group analyses of the cross-sectional studies, the absolute HGS values were higher in the post-BS group compared to the control groups. Of note, although cross-sectional studies were included in this review, these results should be interpreted cautiously, as control groups might not be the best representation of patients at baseline. For instance, the differences in BMI from the control groups (“preoperative”) to the postoperative group of patients were highly variable between the 3 studies analyzed, varying from high differences such as 17.1 kg/m^2^ [46], to minimal differences of 5.3 kg/m^2^ [47].

Age is a very relevant factor to consider in the risk of sarcopenia development. These studies mostly include individuals below 60 years old, meaning additional work is needed to globally investigate the prevalence of sarcopenia in all candidates for BS, including not only muscle mass, but also the analysis of muscle function and muscle quality. Another limitation is that most of the publications described in this review studied a population of mainly female patients, sometimes 100% female. However, this a common bias and difficult to overcome, since more female patients perform BS [76].

Another variable is the importance of the type of surgery performed. Coral et al. did not find differences muscle function changes between SG and RYGB longitudinally [49]. By contrast, de Oliveira et al. showed that patients after SG had better results at the GS test when compared to RYGB patients (1.2 ± 0.3 m/s vs. 0.9 ± 0.1 m/s) [50]. However, other relevant characteristics were statistically different between these groups, namely the BMI at baseline (pre-surgery) and the proportion of weight loss [50]. Another cross-sectional study found similar HGS and SPPB scores between SG and RYGB, despite the higher percentage of weight loss in RYGB [77], while a systemic review with meta-analysis of 52 studies (total of 2270 individuals) also found that when normalized to the total weight loss achieved, the degree of the muscle mass loss was independent of the type of surgery performed [78].

## Conclusions

Sarcopenia, as measured by physical performance, muscle mass, and strength as defined by the EWGSOP, is understudied in persons living with obesity, especially after bariatric and metabolic surgery. We found that the prevalence of pre-existing sarcopenia in patients living with obesity varies significantly due to differing assessment methodologies and definitions. Even if most studies report reductions in skeletal muscle or lean mass after surgery, improvements in muscle functionality (albeit not in muscle absolute strength) are achieved along body weight loss. These changes were predominantly independent of the type of surgery performed.

Although this literature review highlights the relationship between sarcopenia and bariatric and metabolic surgery, there is still insufficient information to reach robust conclusions on the prevalence of sarcopenia and long-term effects after the surgery in patients over the age of 60 and in male patients. The studies discussed are, to the best of our knowledge, the ones that best-described changes in the complete set of sarcopenia-related parameters, namely muscle mass, strength, and function, after bariatric and metabolic surgery through EWGSOP accepted definitions, and so gives the first overview of the subject. Finally, our study was not a systematic review, and some pertinent published studies may have been inadvertently omitted.

## Data Availability

No datasets were generated or analysed during the current study.
